# Conflicting Roles of Connexin43 in Tumor Invasion and Growth in the Central Nervous System

**DOI:** 10.3390/ijms19041159

**Published:** 2018-04-11

**Authors:** Miaki Uzu, Wun Chey Sin, Ayaka Shimizu, Hiromi Sato

**Affiliations:** 1 Laboratory of Clinical Pharmacology and Pharmacometrics, Graduate School of Pharmaceutical Sciences, Chiba University, Chiba-shi, Chiba 263-8522, Japan; muzu@ncc.go.jp (M.U.); color.0v0.flower.182@gmail.com (A.S.); 2 Division of Cancer Pathophysiology, National Cancer Center Research Institute, Chuo-ku, Tokyo 104-0045, Japan; 3 Department of Cellular and Physiological Sciences, Life Sciences Institute, University of British Columbia, Vancouver, BC V6T 1Z3, Canada; wc.sin@ubc.ca

**Keywords:** connexin43, central nervous system, glioma, astrocyte, blood–brain barrier, neurovascular unit

## Abstract

The tumor microenvironment is known to have increased levels of cytokines and metabolites, such as glutamate, due to their release from the surrounding cells. A normal cell around the tumor that responds to the inflammatory environment is likely to be subsequently altered. We discuss how these abnormalities will support tumor survival via the actions of gap junctions (GJs) and hemichannels (HCs) which are composed of hexamer of connexin43 (Cx43) protein. In particular, we discuss how GJ intercellular communication (GJIC) in glioma cells, the primary brain tumor, is a regulatory factor and its attenuation leads to tumor invasion. In contrast, the astrocytes, which are normal cells around the glioma, are “hijacked” by tumor cells, either by receiving the transmission of malignant substances from the cancer cells via GJIC, or perhaps via astrocytic HC activity through the paracrine signaling which enable the delivery of these substances to the distal astrocytes. This astrocytic signaling would promote tumor expansion in the brain. In addition, brain metastasis from peripheral tissues has also been known to be facilitated by GJs formed between cerebral vascular endothelial cells and cancer cells. Astrocytes and microglia are generally thought to eliminate cancer cells at the blood–brain barrier. In contrast, some reports suggest they facilitate tumor progression as tumor cells take advantage of the normal functions of astrocytes that support the survival of the neurons by exchanging nutrients and metabolites. In summary, GJIC is essential for the normal physiological function of growth and allowing the diffusion of physiological substances. Therefore, whether GJIC is cancer promoting or suppressing may be dependent on what permeates through GJs, when it is active, and to which cells. The nature of GJs, which has been ambiguous in brain tumor progression, needs to be revisited and understood together with new findings on Cx proteins and HC activities.

## 1. Introduction

The gap junction (GJ) protein connexin (Cx) 43 forms intercellular channels permitting the passage of small ions and signaling molecules between adjacent cells [1,2]. In particular, connexin43 (Cx43), an ubiquitous isoform, is deeply involved in regulating cell functions mainly via three kinds of approaches; (1) the channel function of the GJ as an intercellular junction; (2) the “hemichannel (HC)” permitting paracrine communication between the cytosol and the extracellular environment [3,4]; and (3) the non-channel function via its C-terminal where intermolecular interaction occurs.

The oncogenicity of a tumor cell depends on its proliferative capacity, motility and viability. Due to the nature of Cx that functions at the cell surface, Cx is associated with all of these cellular phenotypes. Induction of inflammatory signaling, such as the treatment of anti-cancer agents and exposure to cytokines, also modulates Cx functions, intra- and extra-cellularly. Therefore, Cx can be a target of drug development; however, whether they promote exacerbations or support clinical improvement depends on the surrounding environment: which cell (or molecule) the Cx is communicating with, or by which structure Cx exerts its function.

The pivotal role of Cx43 in promoting tumor progression has been previously overlooked because downregulation of Cx43-mediated intercellular communication is normally associated with increased malignancy in tumor cells [5,6]. Indeed, for a long time, it was believed that the GJ formed by Cx43 has mostly tumor suppressive effects [5,6,7], such as its role in anti-proliferation [8,9], anti-metastasis [10], and pro-apoptosis [11,12,13,14,15]. In contrast, emerging evidence indicates that Cx43 serves a facilitative role in tumorigenesis [5,16], especially in advanced stages [7]. Additionally, it has been demonstrated that Cx43 allows the tumor cells to hijack programs that are part of normal tissue development [17]. 

In this review, we focus on the paradoxically unique characteristics of Cx43 in cancer pathology of the central nervous system (CNS), in particular, gliomas, which are aggressive brain tumors, and metastatic cancers that invade the brain from the peripheral tissues in light of recent findings.

## 2. The Role of Connexin43 (Cx43) in Tumors of the Central Nervous System (CNS)

In the CNS, several Cx molecules have been detected: Cx30, Cx32, Cx36 and Cx43 in neurons; Cx30, Cx40, Cx43, and Cx45 in astrocytes; Cx32, Cx36 and Cx43 in microglia; and Cx26, Cx32, Cx29, Cx36, and Cx47 in an oligodendrocyte [18,19,20]. Although they were observations from rodents, Cx43 is one of the subtypes that showed high conservation in the majority of positions shown to be important residues for channel oligomerization, gating, permeation and docking [21], and is the major subtype in the CNS. GJs formed by Cx43 are often observed between astrocytes, and cell junctions with heterologous cells have also been reported. The paradoxical roles of this molecule are highlighted in Table 1.

### 2.1. Involvement of Cx43 in Inflammatory Responses in CNS Cancer

Inflammation is associated with cancer initiation and progression [33]. The inflammatory response induced in the host tissue by the tumor cells accounts for the major signaling pathways hijacked by tumor cells to promote their own survival and expansion [34]. In the brain, one prominent feature of glioma pathology is extensive astrogliosis, an inflammatory response that involves the recruitment of reactive astrocytes around gliomas and brain metastases [35,36,37]; it is often difficult to distinguish glioma cells from reactive astrocytes clinically [35]. Reactive astrocytes express increased levels of glial fibrillary acidic protein (GFAP) intermediate filaments [38,39] and pro-inflammatory cytokines [40]. Although many signaling proteins are upregulated in activated astrocytes, the significance of these proteins, and whether they can be exploited to control brain diseases, remains to be explored. 

Cx43 is the most abundant Cx isoform in adult astrocytes [41,42]; it has been shown to be upregulated in reactive astrocytes induced by various brain pathologies including brain ischemia and epilepsy [18,43,44,45,46,47,48,49]. Similarly, it was demonstrated that reactive astrocytes with enhanced GFAP expression showed an upregulation of Cx43 in a mouse model consisting of intracranial syngeneic implantation of GL261 glioma cells [50]. Interestingly, Cx43 staining was increased in low grade gliomas [24]. A query on the provisional TCGA revealed upregulation of Cx43 in low-grade gliomas may be correlated with a shorter disease-free period. These observations contrast with the prevailing knowledge that carcinogenesis is usually accompanied with a reduction in GJIC [5,6].

In addition to glioma, there are also cancer cells in CNS that originate from outside the brain due to metastasis [28]. After metastatic cells survive the harsh environment of blood flow, they have to go beyond several layers of cells to escape from the blood vessels and infiltrate the brain. The penetration of the first layer is facilitated by their interaction with cerebral endothelial cells, which are damaged by tumor cells via activation of several signaling pathways. The small GTPase (Rho/ROCK) pathway, the PI3K/Akt pathway, and the TGF-β pathway are considered to be involved in this interaction [51]. A single metastatic cancer cell that dislodges from the blood vessels would probably be targeted by the exclusion network of the CNS that includes the resident immune cells—microglia. In addition, the generation of plasmin from neuron-derived plasminogen by astrocyte that cleaves Fas ligand (FasL) will target the cancer cell [52] and thereby induce a paracrine death signal in cancer cells. The interaction of a cancer cell with astrocytes is a possible mechanism to escape this attack. Integrins and GJs are attracting attention as molecules that mediate these heterogeneous cell–cell interactions. 

The question remains on the mechanism by which Cx43 contributes to CNS cancer progression. One possibility is the opening of unpaired Cx43 HCs [53,54,55] under pathophysiological conditions that usually results in the release of bioactive molecules including ATP [56] and provides a possible route for Cx43 to participate in the pro-inflammatory responses known to promote tumor progression [57]. The release of ATP leads to the activation of purinergic receptors in microglia, and a prominent feature of glioma pathology [34]. Microglia have a critical role in modulating the microenvironment by secreting cytokines that affect tumor growth and migration [58]. There is evidence to suggest that HC activity can propagate apoptosis in glioma cells [59]. Therefore, there is a possibility that HC-mediated ATP release will promote the interaction between reactive astrocytes and microglia and affect cancer progression. 

### 2.2. Opposing Roles of Cx43 in Tumor Survival and Invasion

Over-expression of Cx43 has been reported to promote glioma migration in a channel-dependent manner [60,61,62], especially in the presence of normal stromal cells such as astrocytes [60,61,62,63]. Cx43-mediated GJIC appears to alter astrocyte morphology in an in vitro co-culture of glioma cells with astrocytes [62,64], suggesting that bi-directional signaling exists between these cells. However, it remains unclear how these interactions modify the invasive properties of glioma cells in vivo. Co-culture of U87MG human glioma cells with normal human astrocytes enhanced the invasive behavior of the glioma cells [25]. It was further demonstrated using chemical inhibitors, siRNAs, and a channel defective Cx43 mutant, Cx43T154A, that functional glioma–glioma GJs suppressed glioma invasion, while glioma–astrocyte and astrocyte–astrocyte GJs promoted glioma invasion [25]. Therefore, migration and invasion are enhanced with low homocellular glioma GJIC and high heterocellular glioma–astrocyte GJIC. Cx43 is also known to promote migration through channel-independent mechanisms; it strengthens adhesive connections between glioma cells and astrocytes via its extracellular loops [60,61,65,66]; and it regulates cytoskeletal dynamics via its carboxy (C)-terminal tail [67,68,69]. The C-tail of Cx43 interacts with various intracellular signaling molecules [12,68,70,71] and serves a role in channel gating [44,72,73].

In addition to its potential role in tumor invasion, Cx43 has been implicated in regulating cell death. One report indicated that Cx43 increased glioma cell resistance to apoptosis by a channel-dependent mechanism [74]. It was also demonstrated that Cx43 increased the resistance of human glioma cells to temozolomide treatment by modulating the mitochondrial apoptosis pathway [27]. On the other hand, it was observed that Cx43 interacted with Bax in the vicinity of the cell membrane, and by promoting its mitochondrial transition, it regulated the apoptotic signal of the cell and enhanced sunitinib susceptibility [12]. 

**Figure 1:**
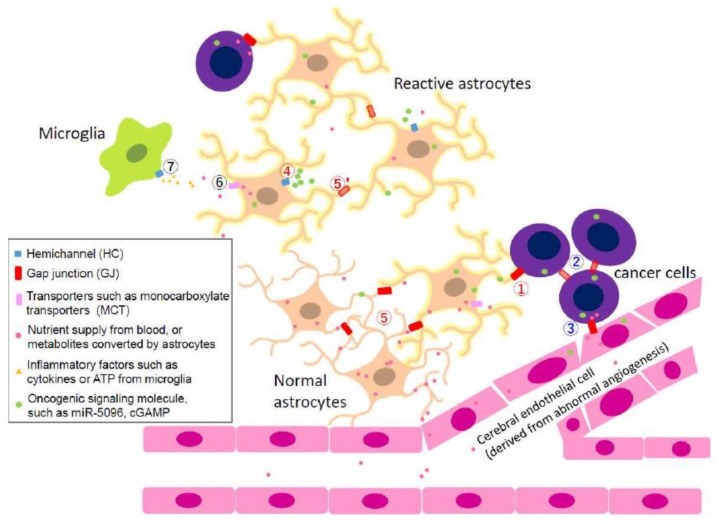
The microenvironment of glioma or metastatic cancers in central nerve system (CNS). Reactive astrocytes have increased Cx43 and GFAP levels, while PTEN, a well-known tumor suppressive protein, is decreased. Cerebral endothelial cells (CECs) also participated here as angiogenesis inducing factor and supply nutrients. Microglia increases glucose uptake by astrocytes. Numbers in circle indicate GJ or HC pathways to maintain this environment. Red colored pathways (***1***, ***4***, ***5*** and ***5′***) are common between glioma and metastatic cancers. Blue colored pathways (***2*** and ***3***) are derived from previous reports on glioma (84,101). Black colored pathways (***6*** and ***7***) denotes those around reactive astrocyte in inflammatory condition [3]. Pathway ***1***: GJ between a cancer cell and an astrocyte; it promotes tumor invasion by transferring oncogenic signaling molecules from a cancer cell to an adjacent astrocyte. Pathway ***2***: GJ between glioma cells; down-regulation of GJ promotes invasion of glioma. Pathway ***3***: GJ between glioma and CEC; it promotes angiogenesis. Pathway ***4***: HC activity of reactive astrocyte; it contributes to spreading of oncogenic signaling molecule in the brain. Pathway ***5***: GJ between normal astrocytes; it supports nutrients supply from CECs to CNS cells. Pathway ***5′***: GJ between astrocytes located distal from the tumor; it is mainly down-regulated when HC is upregulated. Pathway ***6***: Transporters expressed in astrocytes located distal from the tumor; they are believed to be upregulated to uptake enough metabolites. Pathway ***7***: HC activity in microglia; it may support increase of glucose uptake in astrocytes, while it decreases spread of glucose between astrocytes.

**Figure 2:**
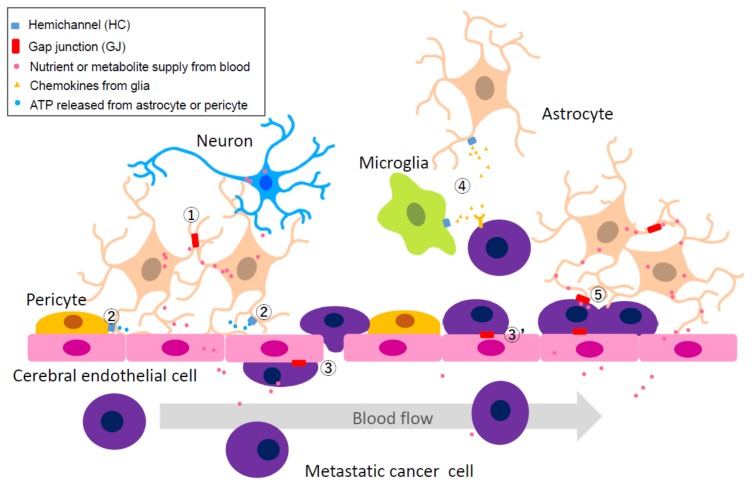
The peri-vascular niche of the tumor microenvironment highlighting the extravasation of metastatic cells. The neurovascular unit includes cerebral endothelial cells (CECs), pericytes, glial cells, and neurons. Metastatic cancer cells utilize the nutrient supply from CECs to CNS cells mediated by the astrocytes. Numbers in circle indicate specific communication pathways. Pathway ***1***: GJ between normal astrocytes; it supports nutrients from CECs to CNS neurons. Pathway ***2***: HC activity of astrocytes and pericytes; it adjusts [Ca^2+^]_i_ concentration in nearby CECs via release of ATP which results in change of blood flow rate. Pathway ***3***: GJ between CEC and metastatic cancer cell inside the capillary; it supports extravascular liberation of cancer cell from blood vessels. Pathway ***3′***: GJ between CEC and metastatic cancer cell located in a pericyte-like location; it protects cancer cells from immune attack by CNS astrocyte and microglia. Pathway ***4***: HC activity of microglia or astrocyte; it supports cancer growth by release of chemokines, although the actual mechanisms by which glial cells promote immune attack or support cancer remains unknown. Pathway ***5***: GJ between metastatic cancer cell located in a pericyte like-location and an astrocyte; it contributes to cancer growth as the first step in brain metastasis.

There is a possibility that Cx43 takes opposite roles depending on the types of cellular interaction. Thus, we next review examples distinguishing whether the GJs mediate effects via homocellular-type or heterocellular-type of interactions in multiple steps of cancer invasion (Figure 1 and Figure 2).

### 2.3. Homocellular Junctions

#### 2.3.1. Glioma Channels in Invasion

The role of homocellular GJIC in glioma migration was highlighted in a recent report using a 3D spheroid migration model that mimics the in vivo architecture of tumor cells to quantify migration changes [75] and found that down-regulation of Cx43 expression in the U118 human glioma cell line increased migration by reducing cell–ECM adhesion, and changed the migration pattern from collective to single cell [75]. More importantly, the ability of a C-terminal truncated Cx43 mutant (TrCx43) to produce migration levels was similar to control cells expressing wild-type (WT) Cx43. In contrast, it was shown that blocking the channel function with a specific mutant (Cx43T154A) and a chemical channel blocker (carbenoxolone (CBX)) increased migration. It suggests that the C-terminal is not mediating migration but GJIC has a more prominent role in migration (Figure 1, Pathway ***2***).

#### 2.3.2. Homocellular Astrocyte Channels in Glioma Invasion

Upregulation of Cx43 has been detected in astrocytomas and peri-tumor parenchyma [76,77,78]. As mentioned in Section 2.1, it was similarly observed that Cx43 immunoreactivity was increased in the brain parenchyma within 100 μm from the edge of the tumor mass [50] in an intracranial mouse model with mCherry-labeled GL261 cells [79,80]. Interestingly, Cx43 co-localizes with podoplanin (PDPN), a glycoprotein implicated to have a role in inflammation [81], in tumor-associated astrocytes [50]. The elimination of Cx43 in host astrocytes surrounding gliomas reduces the invasion of these tumor cells into the surrounding brain parenchyma [24]. Despite robust intercellular communication between astrocytes (Figure 1, Pathway ***5***), no significant increase in HC activity in the peri-tumor region was observed [24], which is in contrast to what is expected from what we know about HC and inflammation (see Section 2.1). In addition, results with the dominant negative channel defective T154A mutant (channel dead Cx43 mutant) demonstrated that Cx43-mediated intercellular communication, between glioma cells and astrocytes, did not seem to be essential for astrocytic Cx43 to mediate its pro-invasive effect in vivo. Taken together, it is revealed an unexplored role of glial Cx43 paracrine signaling that can provide a permissive niche for glioma invasion (Figure 1, Pathway ***4***).

### 2.4. Heterocellular Junctions

#### 2.4.1. Heterocellular GJIC in Glioma Invasion

Investigations on the specific roles of Cx43 in glioma invasion have been hindered by the fact that alteration in tumoral Cx43 levels not only disrupt intra-tumoral homocellular communication, but also perturb the formation of heterocellular channels between glioma cells and astrocytes. In addition, the opposing effects of homocellular and heterocellular GJIC on glioma migration [60] further complicates the role of tumoral Cx43 in glioma progression. As mentioned previously, it was suggested that glioma–glioma GJs attenuate migration while glioma–astrocyte and astrocyte–astrocyte GJs promote glioma invasion [25]. The pro-invasive effects may arise from transfer of oncogenic signaling molecules from glioma cells to adjacent astrocytes via Cx43 (Figure 1, Pathway ***1***), followed by spread of these signals (and/or their downstream effectors) among astrocytes through astrocyte–astrocyte GJs (Figure 1, Pathway ***5***).

The direct passage of small ions and metabolites such as Ca^2+^ [52], ATP [2,4,56], glutamate [82], glucose [83], and peptides [84] through Cx43 channels are well known. However, increasing evidence has demonstrated that Cx43 is permeable to oligonucleotides as long as 24 nucleotides in length [85]. Subsequently, siRNA and microRNA (miRNA) have been shown to exert functional effects on neighboring cells via GJs by using well established chemical inhibitors and reporter proteins such as GFP and luciferase [85,86,87,88,89,90]. Increasing evidence also demonstrate that glioma–astrocyte GJs were permeable to miRNAs [25]. Specifically, miR-5096 was identified to enhance the invasiveness of glioma cells in a Cx43-dependent manner, by using a combination of techniques that include the use of GJ inhibitors and an anti-miR™ that “neutralizes” the action of miR-5096 [25]. The increase of miR-5096 was not due to endogenous upregulation by the astrocytes because the corresponding larger, GJ-impermeable primary miRNA that is the precursor of mature miR-5096 was not detected in the astrocytes [25]. These findings add to recent studies which demonstrate that cancer cells “reprogram” normal stromal cells by miRNAs [22,91]. In this aspect, astrocytes promote brain metastasis by silencing PTEN (phosphatase and tension homology), a protein well known for its tumor suppressive property, via miR-19a using exosomes as a delivery vehicle [92]. On the other hand, it is also known that miR-5096 originating from glioma interacts with human microvascular cerebral endothelial cells (HMEC) through GJ and promotes angiogenesis [26] (Figure 1, Pathway ***3***). Therefore, glioma cells directly influence the gene expression of astrocytes and HMEC and, vice versa, the microenvironment in turn facilitates the invasion of tumor cells.

#### 2.4.2. Heterocellular GJs or HCs: Extravascular Liberation of Metastatic Cancer—Neurovascular Unit Including Cerebral Endothelial Cells (CECs), Pericytes, Glial Cells, and Neurons

For cancer cells to penetrate the CNS, it is necessary to break through the blood–brain barrier (BBB). BBB consists of CECs, pericytes, and glia (Figure 2). One factor that is essential for cancer metastasis is the condition of blood flow. For metastatic cells to undergo extravascular liberation, cells need to remain on the wall in the vascular lumen (Figure 2, Pathway ***3***), therefore it is physically difficult for the infiltration process to occur if the blood flow is too rapid. Substantial evidence suggests that the neurovascular unit facilitates cancer metastasis. High levels of Cx expression are observed at the endfeet of astrocytes that enclose the blood vessels [93,94]. An increase in [Ca^2+^]_i_ in astrocytes is observed during vasodilation or contraction [93,95,96] (Figure 2, Pathway ***2***). The morphological changes of endothelial cells, such as the decision to contract or expand, depends on the nature of the signal that changes extracellular Ca^2+^ concentration. It is an interesting possibility that Cx43 present in the endfeet will affect the perivascular velocity of propagation of this [Ca^2+^]_i_ wave to adjacent astrocytes. It has been suggested that the expression of Cx channels increases the number of endfeets in contact with the CEC and is involved in regulating blood flow [93,97,98]. It is also suggested that HCs are involved in [Ca^2+^]_i_ waves, possibly via ATP release regulating blood flow. Pericytes enclosing CECs have thick basement membrane, but its basement between astrocytes are thin and chemical exchange tends to occur. Furthermore, Cx43 HCs are present on the surface of pericytes and have been demonstrated to play an important role in the pericyte-mediated vascular network communication [99] (Figure 2, Pathway ***2***). Thus, even at the capillary level, the blood flow rate is precisely controlled, and this regulation mechanism may be utilized by cancer cells when they leave the blood vessel and infiltrate the brain.

Metastatic cancer cells usually remain in close proximity to the blood vessels immediately after breaking through the BBB; this perivascular region is not only favored by cancer cells but also is a niche preferred by dormant cells such as neural and glial stem cells [100]. An important progenitor cell proliferation pathway mediated by vascular endothelial growth factor (VEGF) occurs at this location, which is a favorable environment for spreading of some cancer cells [101]. Therefore, the metastatic cancer cells tend to remain attached to the surface of the outer wall of the blood vessel (Figure 2, Pathway ***3***), supported by the presence of GJ formed between them. Regarding the intracerebral infiltration of breast cancer and melanoma cells, GJs consisting of Cx26 and Cx43 have been demonstrated between CECs and invasive cancer cells, and CBX, a GJ inhibitor, was able to decrease the size or number of microcancer colonies in the brain [23]. In addition, there are also microglia around the cerebral vascular unit. It is reported that microglia secrete cytokines and exosomes in the primary tumor of the brain [102,103], which may contribute to proliferation in the brain environment of cancer cells located around blood vessels (Figure 2, Pathway ***4***). It also activates PI3K/Akt signaling and IGF signaling, which in turn promotes angiogenesis in CECs [32]. Further studies are needed to determine to what extent such functions of microglia support extravasation of metastatic cancer in the brain.

#### 2.4.3. Heterocellular GJs: Possibility of Utilizing Metabolic Coupling between Astrocytes and Neurons

There are approximately 86 billion neurons and 85 billion glia (sum of astrocyte, microglia, and other glia) in the whole human brain [104]. Astrocytes residing around neurons play a key role for the maintenance of neuronal excitability: recycling neurotransmitters released into the synaptic cleft, buffering pH or K^+^, and supplying neurons with antioxidants and metabolites [105,106]. GJs and HCs are intimately related to these astrocytic functions (Figure 2, Pathway ***1***).

Recycling of glutamate, the primary excitatory neurotransmitter, is one of the critical roles for astrocytes since overstimulation of glutamic receptors is highly toxic to neurons. To prevent this excitotoxicity, the rapid uptake of glutamate is performed via glutamate transporters, like glutamate transporter 1 (GLT-1) and glutamate aspartate transporter (GLAST) expressed in astrocytes. It is shown that glutamate is able to directly pass through GJs, which is inhibited by the treatment of CBX, and the inhibition of GJs sensitizes neurons to glutamate toxicity [107,108]. These results suggest that the passage of glutamate or its metabolites through GJs may spread a signal among astrocytes to have them support the survival of neurons (Figure 2, Pathway ***1***). In addition, glutamate uptake is accompanied by the co-transportation of Na^+^, which generates a metabolic wave and evokes energy demand [109]. The propagation of this metabolic wave is mediated by ions, and glycolytic metabolites such as glucose and lactate also seem to be involved in the promotion of metabolism. Rouach et al. revealed that a fluorescent glucose derivative 2-[*N*-(7-Nitrobenz-2-oxa-1,3-diazol-4-yl)Amino]-2-Deoxyglucose (2-NBDG) can pass through GJ between astrocytes, and the addition of glucose or lactate stimulates the neuronal activity in the mouse brain, which is abrogated in Cx43-knock out mice [110]. This work suggests that the spread of glycolytic metabolites via astrocytic GJs also contributes to a signal transmission among astrocytes and positively affects neuronal activity. Monocarboxylate transporters (MCTs) are the only known lactate transport carriers, however, a recent study shows that HCs are related to the extracellular release of lactate and this phenomenon enhanced synaptic transmission [111]. Considering these reports, astrocytic GJs and HCs cooperate with each other and contribute to the astrocytic functions to support surrounding neurons. It has also been reported that cytokines secreted from microglia under inflammatory conditions enhance glucose uptake by astrocytes through HCs while reducing glucose diffusion through GJs between astrocytes [3] (Figure 1, Pathway ***4***–***7***), thus the change of activity of HCs and GJs may be involved with the appearance of reactive astrocyte in inflammatory conditions. There is a possibility that a more specific metabolic network may be developed in cancer pathology where inflammatory state occurs throughout the brain tissue.

It is expected that the above astrocyte–neuron metabolic network occurs between astrocytes and invasive cancer cells (Figure 2, Pathway ***5***). Cancer cells from the primary tumor move to the various organs via blood flow. As mentioned in the previous Section 2.1, most of metastatic cancer cells may not survive due to the action of astrocytes by generation of plasmin from neuron-derived plasminogen, which promotes release of membrane-bound astrocytic FasL [52]. Additionally, another target of active plasmin is the L1CAM, an adhesion molecule, which block the interaction between cancer cells and the capillaries [52]. On the other hand, cancer cells that survived the extravasation of blood vessels may utilize the heterocellular metabolic communication to adapt themselves to a new environment. In our preliminary study, significant changes in intracellular metabolome were confirmed in both astrocytes co-cultured with culture supernatant of cancer cells and cancer cells co-cultured with astrocyte culture supernatant, respectively, compared with single cultures. Interestingly, the metabolome did not change if a main metabolite of the pathway is artificially added to the culture system of cancer cells or vice versa. The result suggests that GJs would be the most likely candidate protein to provide continuous and bidirectional intercellular communication between an astrocyte and a cancer cell to induce metabolome change in the cell. 

#### 2.4.4. Heterocellular GJs: Pro-Survival Functions of GJs between Metastasized Cancer Cells and Astrocytes

Although astrocytes have important role to eliminate cancer cells by PA or FasL as mentioned above, they are also able to facilitate tumor survival and proliferation in the right circumstances. For example, brain metastatic melanoma cells are surrounded by reactive astrocytes with morphological changes and expressing high GFAP levels in the brain tissue of either human patients or cancer mouse models [29] (Figure 1, Pathway ***1***); and direct contact with astrocytes confers the drug resistance on melanoma cells, which is inhibited by the treatment of CBX or Cx43-specific siRNA for astrocytes. These data suggest that the direct coupling between a metastasized cancer cell and a reactive astrocyte GJs alters the intracellular survival signals [111], and promotes the survival of cancer cells.

One of the well-known second messengers passing through GJs is Ca^2+^ [55], and apoptosis is induced by the elevation of a [Ca^2+^]_i_ [112]. The previous report shows that astrocytic GJ is involved in the suppression of the rapid increase of [Ca^2+^]_i_ in melanoma cells induced by the treatment of anti-cancer agents, indicating that GJs contribute to sequestering the toxic signal transmitter [29]. Another mechanism by which astrocytic GJs promote brain metastasis was elucidated by Chen et al. who revealed that cyclic dinucleotide cyclic GMP-AMP (cGAMP) is transmitted via GJs from brain metastatic breast and lung cancer cells to astrocytes, which promotes cancer cell growth [31] (Figure 1, Pathway ***1***). 

## 3. Conclusions

Traditionally, GJs were often thought to function mainly in an anti-cancer context. It is now clear that GJ or HC composed of Cx43 are involved in many steps in the progression of brain cancer. HC activity of the astrocyte is proposed to be more suited for “long range” signaling. In contrast, the critical role of GJ is especially strong in metastatic cancers, in which GJs formed between CECs and a metastatic cancer cells is essential for the process of invasion from the outside of the brain through the blood vessel into the brain. Once in the brain tissue, the cancer cells have the opportunity to further integrate by communicating with astrocytes and microglia. These subsequent steps are predicted to be common in primary gliomas and brain metastasis. GJs formed between heterologous cells become an initial step of establishing cancer seeds before their subsequent expansion in the brain tissue. Although substances shown to be communicated between heterologous cells include intracellular secondary messengers such as microRNAs and cGAMP, there is a possibility that a cancer cell receives the gift of a metabolite which is originally used to benefit neurons. Alternatively, toxins such as glutamate or an anti-cancer agent (either an effective drug metabolite (ganciclovir) or stress factor (Ca^2+^)) can flow from cancer cells to astrocytes, which helps to reduce toxicity in cancer cells. The prognosis is expected to depend on the multiple roles played by astrocytes, which make up the majority of glia constituting the brain parenchyma. The specific role of GJs, whether it is cancer suppressive or promoting, depends on the stages of cancer, whether they are undergoing extravasation or proliferation, and the identity of the cell types that make contact with the cancer cells. There has been a report that BBB-permeable GJ inhibitors can suppress brain metastasis in a mouse model [31]; therefore, targeting the direct interaction between metastasized cancer cells and astrocytes may be a prominent therapeutic treatment for brain metastasis. Future identification of pathological targets and technological innovation that enhances the selectivity for targeting (selection of cells, choice of GJ or HC) will improve clinical applicability.

## Figures and Tables

**Table 1:** Summary of the role of Cx43 with specific cell combinations in cancer pathology.

Cell Combination	Form of Cx	Transmitter or Partner Molecule	Effect on Malignant Behavior and Mechanisms Suggested to Be Occurred in Cancer Cells	Ref.
Breast cancer–Osteocyte	HCs	ATP	Released ATP from osteocyte inhibits growth, migration and invasion ability of breast cancer cells	[9]
Mesothelioma–Mesothelioma	Cx molecule (C-terminal)	Src, Bax, JNK	Increasing level of Cx43 in malignant mesothelioma cell enhances sensitivity against cisplatin and sunitinib treatment	[11,12,13]
Leukemic cell–BMSCs	GJ	-	GJ between Cx43- overexpressed BMSCs and leukemic cells induced apoptosis in leukemic cells due to caspase 3/7 activation	[14,15]
Glioblastoma–HMEC	GJ	miR-145-5b	miR-145-5b from HMEC is transferred to glioblastoma (U87 cells) which decrease cancer proliferation	[22]
Colon cancer–HMEC	GJ	miR-145-5b	miR-145-5b from HMEC is permitted to be transferred to cancer cells (SW480 cells) and up-regulated Cx43 expression, which inhibits proangiogenic effect of cancer cells	[23]
Glioma–Glioma	GJ, Cx molecule (extracellular loop and/or C-terminal)	miR-5096	GJ between glioma–glioma has anti-invasive effect	[24,25]
Glioblastoma–HMEC	GJ	miR-5096	mir-5096 from glioblastoma is transferred to HMEC increases proangiogenic effect of glioblastoma	[26]
Glioma–Glioma	GJ, Cx molecule (C-terminal)	Bcl-2, Bax	Increasing level of Cx43 in glioma cell enhances resistance against temozolomide treatmentIncreasing level of Cx43 in glioma cell enhances resistance against temozolomide treatment	[27]
Microglia–Astrocyte	GJ, HCs	IL-1β, TNF-α	Intercellular diffusion of glucose in CNS via GJ composed of Cx43 between astrocytes is downregulated by cytokines secreted from HCs of microglia. Oppositely, when glucose uptake in each astrocyte is increased, it switches the cell to be a reactive astrocyte.	[3]
Glioma–Astrocyte, Astrocyte–Astrocyte	GJ, Cx molecule (extracellular loop and/or C-terminal)	miR-5096	Glioma–astrocyte and astrocyte–astrocyte promotes glioma invasion	[25,28]
Glioblastoma–HMEC	GJ	miR-5096	mir-5096 from glioblastoma (U-87 cells) to HMEC increases proangiogenic effect	[26]
Melanoma–Astrocyte	GJ	-	Direct contact with astrocyte up-regulates invasion of cancer cells and drug resistance	[29]
Breast cancer–Astrocyte, Lung cancer–Astrocyte	GJ		GJ signaling enhances production of cytokines in cancer cells and endothelin in astrocytes, which in turn upregulate AKT/MAPK signaling in breast cancer (MDA-MB-231) and lung cancer (H226) cells to protect from cytotoxicity of chemotherapeutic drugs	[30]
Breast cancer–Astrocyte, Lung cancer–Astrocyte	GJ	cGAMP	cGAMP from metastasized cancer cells to astrocytes induces STING signaling in astrocytes, which in turn stimulate cancer metastasis	[31]
Microglia–Retinal cerebral endothelial cell	HCs		Microgila secretes basigin via activation of PI3K/Akt signaling or IGF signaling that in turn promote angiogenesis in cerebral endothelial cell	[32]

Tumor suppressive effect of Cx43 is in green while those in pink indicates Cx43 works in a tumor promotive manner. Although Table 1 contains Cx43 reports regarding various cell combinations not limited to CNS, it is generally accepted that the fundamental role of connexin is the same in peripheral tissues and the CNS. Bcl2-associated protein X, Bax; BMSCs, bone mallow stroma cells; cGAMP, 2′3′-cyclic GMP-AMP; CNS, central nervous system; DC, dendritic cells; GJ, gap junction; HCs, hemichannels; HMEC, human micro vascular cerebral endothelial cells; Interleukin 1 beta, IL-1β; JNK, c-Jun N-terminal kinase; Src, Proto-oncogene tyrosine-protein kinase Src; TNF-α, Tumor necrosis factor alpha.
